# Molecular Research on Amyotrophic Lateral Sclerosis

**DOI:** 10.3390/ijms232012069

**Published:** 2022-10-11

**Authors:** Luisa Agnello, Marcello Ciaccio

**Affiliations:** 1 Department of Biomedicine, Neurosciences and Advanced Diagnostics, Institute of Clinical Biochemistry, Clinical Molecular Medicine and Clinical Laboratory Medicine, University of Palermo, 90127 Palermo, Italy; 2 Department of Laboratory Medicine, University Hospital “P. Giaccone”, 90127 Palermo, Italy

Amyotrophic Lateral Sclerosis (ALS) is a rare, progressive, lethal, and degenerative disease of motor neurons for which there is no treatment currently available. It exists in two forms, sporadic (90% of cases) and familiar, which is due to mutations in more than 20 genes.

The exact pathophysiological mechanism underlying selective motor neuron degeneration is still unclear. However, the involvement of some pathways has been hypothesized, including oxidative damage, defects in axonal transport, inflammation, and mitochondrial dysfunction. Additionally, diagnosing ALS is challenging and typically takes many months to complete. Thus, there is ongoing research attempting to unravel the cellular mechanisms involved in the development and progression of the disease, explore the role of genetics and other potential risk factors, detect biomarkers, and develop new treatments.

Recently, animal and postmortem studies have suggested a role for selenoprotein P, a selenium-transporter protein, in the pathophysiology of neurodegenerative diseases, including ALS. Urbano et al. first evaluated the possible role of cerebrospinal fluid (CSF) and blood selenoprotein P in the etiology and progression of ALS and Alzheimer’s Disease (AD) in vivo by performing a case-control study [1]. The authors reported altered levels of the biomarker in both ALS and AD, but with a different trend. Thus, further studies to clarify the role of selenoprotein P in neurodegenerative diseases are necessary. Interestingly, it has been suggested that exposure to various environmental factors, including solvents, heavy metals, and pesticides, such as selenoprotein, could be involved in the pathogenesis of ALS by enhancing oxidative damage [Motataianu]. Motataianu et al. described the evidence from the current literature on the relationship among genetics, environmental factors, and oxidative stress occurrence in ALS [2].

Evidence from the literature supports the role of the immune system in ALS progression. Specifically, monocytes expressing the inflammation suppressing active CD11b, a beta2 integrin, could be involved in the regulation of neuroinflammation in ALS patients. Yildiz et al., in a retrospective study including 38 ALS patients and 20 non-neurological controls, showed that CD11b^+^ monocytes predicted shorter survival [3]. Thus, these preliminary findings suggest a possible role of CD11b^+^ monocytes as prognostic biomarkers of ALS.

Another component of the immune system involved in the progression of ALS is the Receptor for Advanced Glycation End Product (RAGE). Recently, Nowicka et al. demonstrated the expression of RAGE and its ligands during the progression of the disease in the transgenic SOD1 G93A mouse lumbar spinal cord [4]. Thus, RAGE and its ligands could represent not only diagnostic but also prognostic biomarkers of ALS.

Interestingly, a role for leptin, a polypeptide hormone secreted primarily by adipocytes involved in the neuroendocrine regulation of food intake, has been hypothesized. Recently, clinical and epidemiological studies suggested the hypothesis that altered leptin levels may contribute to the ALS pathogenesis. Ferrer-Donato et al. explored the mechanisms underlying the relationship between leptin and ALS in experimental models of ALS [5].

Since the global prevalence of ALS is predicted to increase up to 29% by 2040, new therapeutic strategies for blocking or slowing ALS are strongly sought after [6]. Among proposed therapeutic targets, chemical chaperones, such as 4-phenylbutyric acid (4-PBA), which have the potential to restore protein homeostasis and improve cellular function, showed promising results. Alfahel et al. investigated the potential therapeutic effect of two 4-PBA derivatives, N-(2-(1-methylpyrrolidin-2-yl)ethyl)-4-phenylbutanamide (compound 4, C4) and 2-isopropyl-4-phenylbutanoic acid (compound 5, C5) in the mutant SOD^1G93A^ mouse model of ALS [7]. They found that C4 or C5 daily injections in SOD1^G93A^ mice following onset failed to extend the survival of SOD^1G93A^ mice or to improve their motor symptoms. Moreover, they showed that high concentrations of C4 and C5 reached the brain and spinal cord, but only for a short period of time. Thus, these findings suggest that the use of C4 and C5 for ALS should be optimized for more effective results.

Another member of the chaperones family, the TNF-receptor associated protein (TRAP1), a cytoprotective mitochondrial-specific member of the Hsp90 heat shock protein, has been proposed as a potential therapeutic target. TRAP1 antagonizes mitochondrial apoptosis and oxidative stress, regulates the mitochondrial permeability transition pore, and controls protein folding in mitochondria. Clarke et al. showed that shRNA-mediated knockdown of TRAP1 expression in primary murine motor neurons results in mitochondrial dysfunction but does not further exacerbate damage induced by oxidative stress alone [8].

Interesting findings have been achieved by inhibiting histone deacetylase (HDAC). Burg et al. showed that the administration of ACY-738, an inhibitor of HDAC, in the spinal cord of a FUS ALS mouse model improved motor neuron deterioration and survival by restoring metabolic alterations [9].

In conclusion, there is intense, ongoing research on ALS, which is leading to interesting findings [10].

## References

[1] Urbano T., Vinceti M., Mandrioli J., Chiari A., Filippini T., Bedin R., Tondelli M., Simonini C., Zamboni G., Shimizu M. (2022). Selenoprotein P Concentrations in the Cerebrospinal Fluid and Serum of Individuals Affected by Amyotrophic Lateral Sclerosis, Mild Cognitive Impairment and Alzheimer’s Dementia. Int. J. Mol. Sci..

[2] Motataianu A., Serban G., Barcutean L., Balasa R. (2022). Oxidative Stress in Amyotrophic Lateral Sclerosis: Synergy of Genetic and Environmental Factors. Int. J. Mol. Sci..

[3] Yildiz O., Schroth J., Lombardi V., Pucino V., Bobeva Y., Yip P.K., Schmierer K., Mauro C., Tree T., Henson S.M. (2022). The Expression of Active CD11b Monocytes in Blood and Disease Progression in Amyotrophic Lateral Sclerosis. Int. J. Mol. Sci..

[4] Nowicka N., Szymańska K., Juranek J., Zglejc-Waszak K., Korytko A., Załęcki M., Chmielewska-Krzesińska M., Wąsowicz K., Wojtkiewicz J. (2022). The Involvement of RAGE and Its Ligands during Progression of ALS in SOD1 G93A Transgenic Mice. Int. J. Mol. Sci..

[5] Ferrer-Donato A., Contreras A., Frago L.M., Chowen J.A., Fernandez-Martos C.M. (2021). Alterations in Leptin Signaling in Amyotrophic Lateral Sclerosis (ALS). Int. J. Mol. Sci..

[6] Roberts B., Theunissen F., Mastaglia F.L., Akkari P.A., Flynn L.L. (2022). Synucleinopathy in Amyotrophic Lateral Sclerosis: A Potential Avenue for Antisense Therapeutics?. Int. J. Mol. Sci..

[7] Alfahel L., Argueti-Ostrovsky S., Barel S., Ali Saleh M., Kahn J., Azoulay-Ginsburg S., Rothstein A., Ebbinghaus S., Gruzman A., Israelson A. (2022). 4-Phenylbutyric Acid (4-PBA) Derivatives Prevent SOD1 Amyloid Aggregation In Vitro with No Effect on Disease Progression in SOD1-ALS Mice. Int. J. Mol. Sci..

[8] Clarke B.E., Kalmar B., Greensmith L. (2022). Enhanced Expression of TRAP1 Protects Mitochondrial Function in Motor Neurons under Conditions of Oxidative Stress. Int. J. Mol. Sci..

[9] Burg T., Rossaert E., Moisse M., Van Damme P., Van Den Bosch L. (2021). Histone Deacetylase Inhibition Regulates Lipid Homeostasis in a Mouse Model of Amyotrophic Lateral Sclerosis. Int. J. Mol. Sci..

[10] Dilliott A.A., Andary C.M., Stoltz M., Petropavlovskiy A.A., Farhan S.M.K., Duennwald M.L. (2022). DnaJC7 in Amyotrophic Lateral Sclerosis. Int. J. Mol. Sci..

